# Health inequalities in outpatient neurological conditions across a large UK urban population: a retrospective observational study using automated coding

**DOI:** 10.1136/bmjno-2025-001532

**Published:** 2026-05-20

**Authors:** Keira Markey, Robyn Hamilton, Rohan Ahmed, Joyutpal Das, Chris Douglass, Goran Nenadic, Karim Webb, James B Lilleker, David Rog, Monty Silverdale, Rajiv Mohanraj

**Affiliations:** 1 Department of Neurology, Northern Care Alliance NHS Trust Manchester Centre for Clinical Neuroscience, Salford, England, UK; 2 Lancaster Medical School, Lancaster University, Lancaster, UK; 3 Data and Analytics Data Science Team, Northern Care Alliance NHS Trust, Salford, England, UK; 4 Faculty of Biology Medicine and Health, The University of Manchester, Manchester, England, UK; 5 Department of Computer Science, The University of Manchester, Manchester, England, UK; 6 University of Salford, Salford, UK

**Keywords:** CLINICAL NEUROLOGY, EPIDEMIOLOGY, HEALTH POLICY & PRACTICE

## Abstract

**Objectives:**

To use automated coding to identify broad neurological diagnoses and link to sociodemographic data.

**Design:**

Retrospective observational study.

**Setting:**

Tertiary outpatient neurology services covering Greater Manchester and East Cheshire.

**Participants:**

All adult patients attending neurology appointments between 1 January 2018 and 1 November 2024, covering a population of 3.3 million.

**Outcome measures:**

To extract and correctly code outpatient neurological diagnoses from semistructured clinical letters and to identify sociodemographic differences.

**Results:**

Successfully extracted diagnostic data were coded and linked to sociodemographic data for 125 273 unique neurology outpatients. Headache (16.1%, n=26 631) and epilepsy (14.3%, n=24 880) were the most common diagnoses observed. Higher rates were seen from the highest social deprivation for females with functional neurological disorder (age-standardised rate ratio (ASRR) (95% CI) 1.78 (1.73 to 1.83)), headache (ASRR (95% CI) 1.64 (1.61 to 1.68)) and males with epilepsy (ASRR (95% CI) 1.36 (1.32 to 1.39)). Females from lower social deprivation were observed at higher rates with demyelination/inflammation (ASRR (95% CI) 1.34 (1.23 to 1.45)). Ethnicity was missing for 16.5% (n=17 523), but Asian, black and mixed ethnicities had lower rates of clinic attendance compared with white.

**Conclusions:**

Automated coding of outpatient neurology data can reveal diagnostic patterns and health disparities, providing insights not previously available at scale. These data offer a powerful tool to support service planning, resource allocation and population-level research.

**WHAT IS ALREADY KNOWN ON THIS TOPIC**

- A 2010 prospective multicentre study, in Scotland, narratively recorded neurological diagnoses for 3781 patients over 15 months. Between 2016 and 2019, a single National Health Service neurology clinic retrospectively assigned diagnostic categories for 1951 patients, manually. Automated outpatient diagnostic coding has been used in two Scottish endocrinology clinics between 2018 and 2019 for 1870 patients.

**WHAT THIS STUDY ADDS**

- To our knowledge, this is the largest automated coding of diagnoses from outpatient neurology clinics covering a large conurbation of Greater Manchester and East Cheshire, United Kingdom (UK), consisting of a population of 3·3 million people.

**HOW MIGHT THIS STUDY AFFECT RESEARCH, PRACTICE OR POLICY**

- Outpatient diagnostic coding would enable disease burden identification, high-risk groups and allow for targeted, equitable and preventative health systems.

## Background

 Structured recording of outpatient data in the United Kingdom (UK) National Health Service (NHS) is neither currently mandatory, nor reimbursed. Consequently, a wealth of patient data is not effectively captured and the potential to evaluate the healthcare needs, inequalities and access of the local population are missed. This is particularly important for outpatient-based specialities such as neurology, rheumatology and dermatology.

Unlike inpatient activity, where coding is linked to financial reimbursement, funding for outpatient services was historically linked to attendance types (e.g. new or follow-up) and specialty, via the NHS payment scheme.^1^ More recently, block contracts (fixed payments regardless of activity) have been introduced, further reducing motivation to improve coding. Given the lack of financial incentives, no clear national standardised coding and clinic time pressures, outpatient diagnostic and procedural coding data collection is missed.^2^

Outpatient consultations in the NHS often generate clinical correspondence in the form of an unstructured (free-text) or semistructured letter sent to the primary care physician or other referrer. Key information in the outpatient letters includes the diagnosis and agreed management plan. The large-scale manual review of historic outpatient letters to extract information is not practically feasible, although this has been done in single clinics.^3^ Thus far, automated extraction of free text data using natural language processing (NLP) is not widely adopted. Modern NLP techniques use deep learning to understand nuances of human language, enabling greater accuracy than earlier rule-based techniques.

Several NLP tools already exist for clinical use, including Medical Concept Annotation Toolkit (MedCAT),^4^ clinical Text Analysis and Knowledge Extraction System (cTAKES)^5^ and Clinical Language Annotation, Modelling, and Processing Toolkit (CLAMP).^6^ In neurology, machine-learning techniques have started to be employed in stroke^7 8^ to extract comorbidity data and for stroke severity estimation, and in epilepsy^9^,^11^ to extract relevant condition-specific information such as seizure frequency.

MedCAT was developed by the Cogstack team^4 12^ to extract and link free-text diagnosis from a clinical letter to standardised ontologies such as Systematic Nomenclature of Medicine Clinical Terms (SNOMED-CT). It has been deployed in several settings, including University College London Hospitals where it was used to process over 30 million records.^13^

Previous studies looking at outpatient coding in the UK have been small. A 2010 prospective multicentre study in Scotland narratively recorded neurological diagnoses for 3781 patients over 15 months.^14^ Between 2016 and 2019, a single NHS neurology clinic retrospectively manually assigned diagnostic categories for 1951 patients.^3^ While automated outpatient diagnostic coding has been used in two Scottish endocrinology clinics between 2018 and 2019 for 1870 patients.^15^

The aim of this study was to extract population-level clinical data from semistructured neurology outpatient letters, in a large urban conurbation of Greater Manchester (GM). We developed and applied NLP tools to automatically code diagnosis and to link these to sociodemographic data. We then intended to explore the influence patient demography and geography had on clinic attendance. This is the first published use of open-source NLP methods for automated coding in outpatient neurology, and to our knowledge, the data extracted form the largest outpatient dataset of coded neurological diagnoses and associated demographic information in the UK, to date.

## Methods

### Study population

The Manchester Centre for Clinical Neurosciences (MCCN), based at Northern Care Alliance (NCA) NHS Trust, is the sole provider of outpatient-based secondary and tertiary neurological services for around 3.3 million people in the GM and East Cheshire regions of the UK. GM is a metropolitan county made up of ten boroughs (Bolton, Bury, Manchester, Oldham, Rochdale, Salford, Stockport, Tameside, Trafford and Wigan) with a population of 2.9 million people. Neurological services are delivered in a ‘hub and spoke’ model, via the neuroscience centre at Salford Royal Hospital, and 13 district general hospitals in the region.

All outpatient clinic letters are available on a single electronic health record system. Patients who attended general and specialist neurology outpatient clinics provided by MCCN between 1 January 2018 and 1 November 2024 were identified from the Patient Administration System (PAS) using specific neurology clinic codes. Since 2015, outpatient clinic letters at MCCN are required to use a semistructured format, with ‘Diagnosis’ or ‘Reason for visit’ listed at the top. Letters not relating to an outpatient clinic encounter with a patient (eg, results letters, multidisciplinary team meetings or ‘advice and guidance’) were excluded from the analysis. The final dataset included the most recent outpatient clinic letter for 1 25 273 patients. For the purposes of analysis, only patients residing within GM were included. East Cheshire was excluded because of part coverage by other providers. The GM patient cohort included a total of 1 05 936 patients over 18 years old.

### Methodology

**Figure 1:**
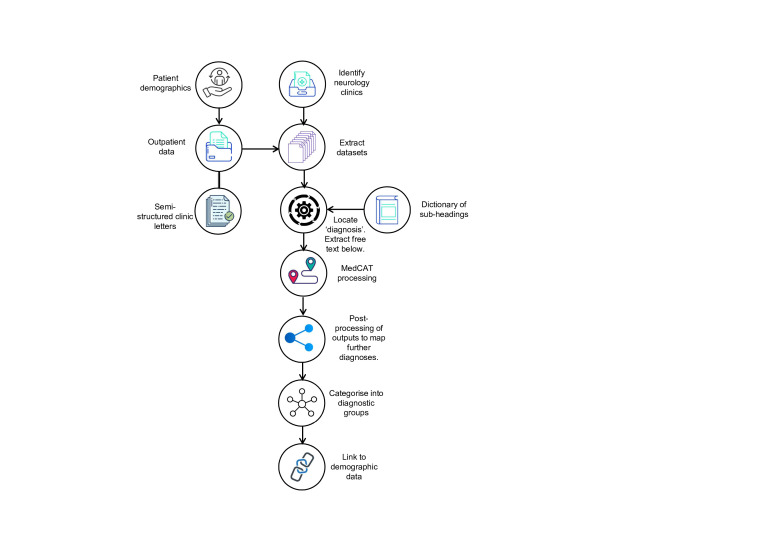
Automated pipeline. Clinic letters were located through EHR. The last clinic appointment letter for each patient was used to extract diagnoses. Using a previously developed dictionary of letter subheadings (n=~3000), different sections of the letter were automatically identified, located and annotated, and the text within each section was extracted. Headings used to denote diagnoses (eg, ‘Diagnosis’, ‘Reason for attendance’, ‘Epilepsy Classification’) were then selected. Free-text diagnoses were processed through MedCAT (code available at GitHub under the Apache 2·0 Licence)^12^ to map each diagnosis to SNOMED-CT. Diagnoses that were not mapped by MedCAT, including abbreviations and acronyms, were reviewed by clinicians and additional post-processing was added to the pipeline. Diagnoses were linked to the Patient Administration Service (PAS) demographic data. PAS is highly reliable for core patient identifiers such as name, date of birth, address (validated via systems like the Personal Demographics Service), but less complete and consistent for optional or self-reported details such as ethnicity. EHR, electronic health record; SNOMED-CT, Systematic Nomenclature of Medicine Clinical Terms.

An automated pipeline (figure 1) was developed at the NCA NHS Foundation Trust to extract patient lists from preidentified neurology clinic codes and demographics from the PAS. The outputs were categorised into diagnostic headline groups as previously described^2 14^ with these diagnostic groups converted into binary columns within the dataset, excluding repetitive diagnoses within each headline group (eg, focal epilepsy, seizure) for each patient.

### Validation

As an initial ‘proof of principle’, seven general neurology clinic’s outpatient letters (n=200), over a period of a month (June 2023), were analysed had all diagnoses extracted then manually reviewed by clinicians. Generic diagnoses from longer free-text expressions proved difficult to extract (eg, extracting ‘hypertension’ only from ‘idiopathic intracranial hypertension’).

After further refinement, a second version of the pipeline was conducted using the same seven clinic codes between 1 January 2018 and 31 December 2023, generating a list of 6194 patients. Excluding patients who did not attend, the dataset reduced to 4549 patients. Diagnoses were extracted for 4380 (96%). Clinicians verified the extracted diagnosis from a 5% random sample. The output from MedCAT produced 3025 SNOMED-CT diagnoses. For data analysis, the dataset was further sorted into larger ‘headline’ category groups (table 1) as per the coding scheme proposed by Biggin and colleagues.^2^ The final validation of the pipeline was performed on patients from the original seven general neurology clinic codes between 1 January 2025 and 31 January 2025, producing 5030 unique patient identifiers. A subset of 210 patients was sampled randomly, and diagnoses were manually extracted by blinded clinicians, assigned to a headline category and compared with the automated output. Analysis was performed on a *per letter* basis, where if multiple mentions of the same diagnosis (eg, focal epilepsy, epilepsy, seizures) were extracted for the same patient, all were automatically aggregated into a single diagnosis. Patients could have multiple different diagnoses as shown in table 1 and online supplemental appendix Figure 1. Precision, recall and the F1 score were determined. Precision represents the proportion of positive predictions that are true, whereas recall is the proportion of actual positive cases. The F1 score is determined by:


$$F1=\frac{\left(2\times Precision\times Recall\right)}{\left(Precision+Recall\right)}$$

**Table 1:** List of categorised diagnoses extracted from clinical correspondence of 98 559 patients at the Manchester Centre for Clinical Neurosciences between 1 January 2018 and 30 November 2024

Diagnosis category	N	%
Headache	23 263	16.96
Epilepsy/seizure	20 724	15.11
Movement disorder	14 347	10.46
Neuromuscular disorder	13 679	9.97
Functional/psychological disorder	13 648	9.95
Suspected neurological diagnosis*	9514	6.94
Demyelination/inflammation	6454	4.71
Spinal degenerative disease	5462	3.98
Non-neurological disorder	3313	2.42
No definite neurological diagnosis made	489	0.36
Other†	26 280	19.16
Total	137 173	

* ‘Other’ refers to patients who diagnoses did not fit into the broader categories, eg, cranial nerve palsies, meningitides, etc.

† ‘Suspected neurological diagnosis’ refers to patients who had a neurological symptom (eg, dysphagia) listed under the diagnosis section of the outpatient letter but without a confirmed diagnosis.

### Analysis

Occurrences of each neurological disease category were analysed for lower layer super output areas (LSOA), which are small geographical areas of around 1–3000 people and defined by the UK Office for National Statistics.^16^ Each neurological disease category was analysed by age, sex and indices of multiple deprivation (IMD) quintiles (1 represents the most deprived and 5 represents the least deprived) from 2019 census data^17^ using R. IMD is a score of relative deprivation based on the LSOA of an individual that measures seven domains, including: income, employment, education, housing, health, crime and environment. Generalised regression models were used to assess significant differences in groups using negative binomial for count data, depending on the data dispersion. ORs were used for binary outcomes while rate ratios (RRs) were used for counts offset by population based on age, sex and LSOA distributions. As the neurology service is adult-only, age was split into clinically relevant categories: 18–39, 40–59, 60–79 and 80+ years. Reference groups for RR comparisons included IMD quintile 1, ages 18–39, females and white ethnicity. Reference categories were chosen as the largest groups (18–39 years, white ethnicity and IMD quintile 1) to provide a stable baseline and minimise variance for comparison interpretation. Age-standardised RRs (ASRRs) were calculated using indirect standardisation to account for differences in age structures, sex and IMD between the clinic and GM population.

## Results

Using a previously unseen dataset, the final validation of the automated pipeline used to extract diagnoses demonstrated precision of 90.6%, recall of 90.0% and an F1 score of 90.3%. F1 scores over 90% are considered comparable to interclinician acceptance and are potentially appropriate for clinical service and evaluation.^11^ A similar study, extracting epilepsy data from outpatient letters, had precision of 94.1%, recall of 95.3% and an F1 score of 94.7% for diagnostic accuracy.^10^ The higher score likely reflects the more select diagnoses used (epilepsy only), while our study includes all neurological diagnoses.

A total of 1 05 936 patients were included in the final dataset. No diagnosis was mapped in 7357 (6.95%) patients. From the remaining 98 579 patients, a total of 137 173 neurological diagnoses were mined (online supplemental appendix Figure 1).

Headaches (n=23 263, 16.96%), epilepsy/seizures (n=20 724, 15.11%), movement disorders (n=14 347, 10.46%), neuromuscular disorders (n=13 679, 9.97%) and functional neurological disorders (FND) (n=13 648, 9.95%) were the most frequent diagnostic categories captured (table 1). For 9514 (6.94%) patients, a neurological symptom (eg, dysphagia) was listed as a diagnosis and so reassigned to the ‘suspected neurological diagnosis’ category.

**Figure 2:**
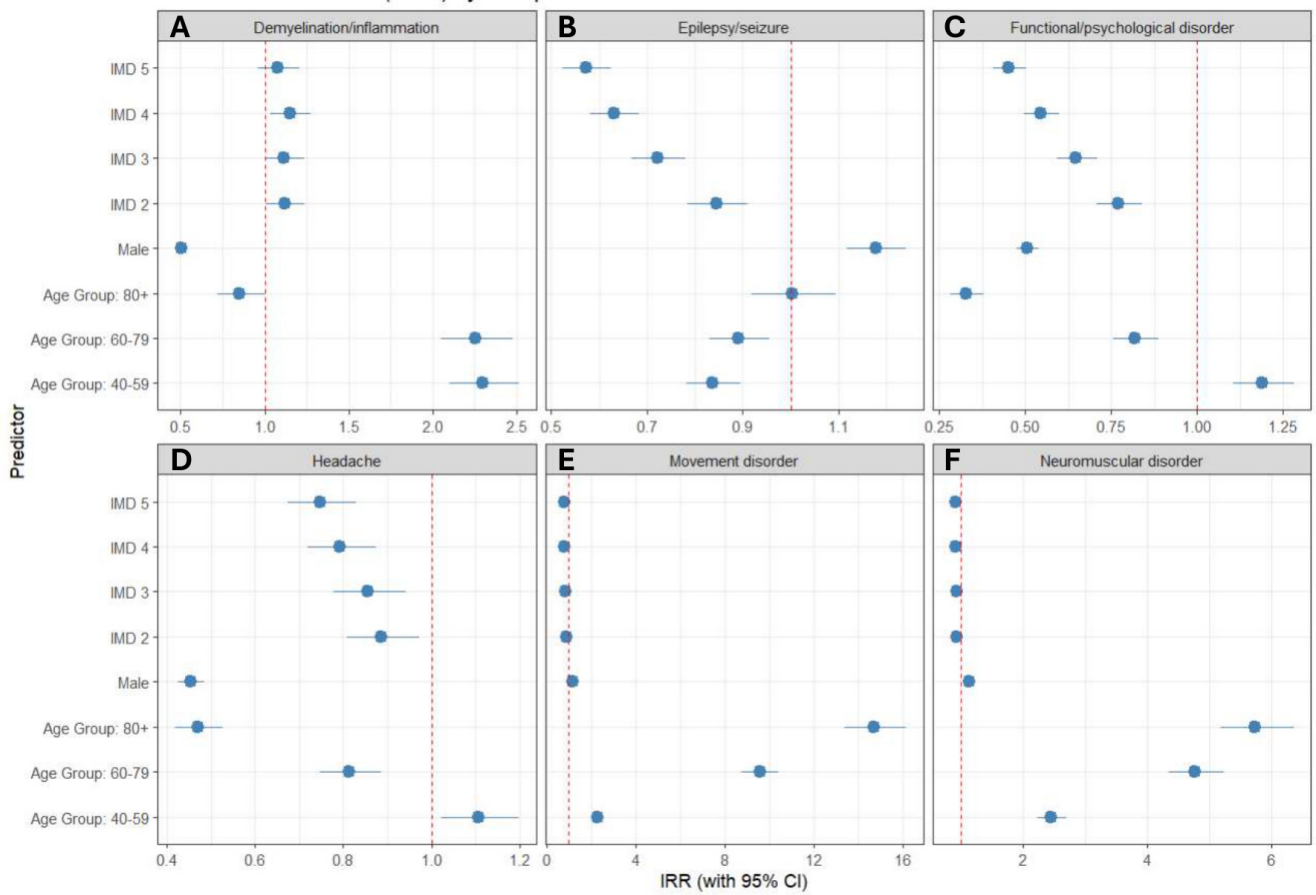
Forest plots showing rate ratios (RR) for the top six diagnostic categories and age, IMD and sex. (A) Demyelination/inflammation. Increased likelihood of being seen in clinic if 40–59 and 60–79 years old, from less deprived backgrounds (higher IMD) but less likely if male or over 80 years old. (B) Epilepsy. Increased likelihood of being seen in clinic if male or from the most deprived background. A lower likelihood of being seen in clinic if 40–79 and from IMD 2–5. (C) Functional/psychological disorder. Increased likelihood if younger (18–39 and 40–59), the most deprived and female. They are less likely with increasing age and increasingly less deprived (IMD 2–5). (D) Headache*.* More likely to be seen in clinic from the most deprived, younger (18–59), and female. Less likely with increasing age and less deprived (IMD 2–5). (E) Movement disorder. More likely to be seen with increasing age. There is no significant difference for IMD or sex. (F) Neuromuscular disorders*.* More likely to be seen with increasing age. There is no significant difference for IMD or sex. IMD, indices of multiple deprivation.

Epilepsy, headache and FND are seen more frequently in the younger adult population in clinic (median age 45) (table 2). Demyelinating/inflammatory disorders are seen more in middle-aged groups but less in the older groups (median age 53). Movement disorders (median age 70) and neuromuscular disorders (median age 63) are seen more frequently in older groups in clinic (figure 2 and online supplemental appendix Figure 2).

**Table 2:** Demographics of headline neurological disorders in outpatients in Greater Manchester

	Headache	Epilepsy/seizure	Movement disorder	Neuromuscular disorder	Functional/ psychological disorder	Demyelination/ inflammation	Spinal degenerative disease	Other
Age (years)								
Median (LQR-UQR)	44 (33–57)	46 (31–61)	69 (58–77)	61 (48–73)	45 (33–57)	53 (41–64)	59 (48–71)	62 (45–74)
Ethnicity (% (n))								
White	68.3 (15 894)	75.0 (15 549)	78.1 (11 209)	79.4 (10 854)	75.1 (10 255)	84.7 (5464)	79.0 (4316)	76.9 (20 213)
Unknown	19.9 (4634)	15.8 (3263)	14.6 (2092)	11.8 (1612)	16.3 (2221)	5.6 (363)	11.9 (650)	15.4 (4040)
Asian	6.7 (1560)	5.1 (1050)	4.6 (660)	4.9 (674)	4.4 (602)	4.9 (318)	5.5 (299)	4.3 (1125)
Black	2.0 (462)	1.7 (351)	1.1 (154)	1.7 (235)	1.3 (178)	1.9 (120)	1.5 (84)	1.5 (395)
Mixed	1.3 (290)	1.2 (239)	0.6 (85)	0.9 (122)	1.4 (186)	1.3 (86)	0.9 (50)	0.8 (206)
Other ethnic group	1.8 (423)	1.3 (272)	1.0 (147)	1.3 (182)	1.5 (206)	1.6 (103)	1.2 (63)	1.2 (301)
Sex (% (n))								
Female	71.5 (16 628)	47.9 (9936)	48.5 (6963)	49.2 (6727)	68.1 (9293)	67.5 (4359)	55.6 (3038)	48.8 (12 822)
Male	28.5 (6635)	52.1 (10 788)	51.5 (7384)	50.8 (6952)	31.9 (4355)	32.5 (2095)	44.4 (2424)	51.2 (13 458)
**Total**	23 263	20 724	14 347	13 679	13 648	6454	5462	26 280

Ethnicity was categorised using standard NHS classifications: White, Mixed, Asian, Black and Other ethnic group. The ‘Other ethnic group’ category included Arab and ethnicity who did not fall into the White, Black, Asian, Mixed ethnicity categories. Patients with missing data were coded as Unknown. Those with no definite neurological diagnosis were excluded from this table (see online supplemental appendix Table 1 for full results).

LQR, lower quartile range; UQR, upper quartile range.

The patient population attending the clinic was predominantly of white ethnicity, who were over-represented compared with population proportions in GM (n=78 400, outpatient vs GM: 88.7% vs 81.3%, p<0.0001) (online supplemental appendix Figure 3). Asian, black and mixed ethnicity patients were under-represented relative to the GM population proportions (outpatient vs GM: Asian 6.2% vs 11.3%, black 2.0% vs 3.6%, mixed 1.3% vs 1.9%; p<0.0001) (online supplemental appendix Figure 3). We note that the ethnicity data were missing for a large number of patients (n=17 523, 16.5%) affecting analysis. In adjusted negative binomial models, patients of Asian ethnicity were significantly less likely to attend clinic when presenting with demyelination/inflammation (RRs (95% CI) 0.36 (0.31 to 0.42), p<0.001), epilepsy/seizure (0.40 (0.35 to 0.46), p<0.001), FND (0.36 (0.31 to 0.41), p<0.001), headache (0.58 (0.50 to 0.68), p<0.001), movement disorder (0.37 (0.33 to 0.42), p<0.001) and neuromuscular disorders (0.38 (0.34 to 0.43), p<0.001). Similar patterns observed for patients of black ethnicity were also less likely to be seen with demyelination/inflammation (RRs (95% CI) 0.43 (0.34 to 0.54), p<0.001), epilepsy/seizure (0.41 (0.35 to 0.50), p<0.001), FND (0.30 (0.24 to 0.38), p<0.001), headache (0.59 (0.48 to 0.72), p<0.001), movement (0.27 (0.21 to 0.33), p<0.001) and neuromuscular disorders (0.44 (0.37 to 0.52), p<0.001). Mixed ethnicity showed similar reduced likelihoods for clinic attendance across most neurological conditions (online supplemental appendix Figure 4).

Overall, patients seen in GM outpatient neurology clinics were most frequently from the most deprived areas and were proportionally higher compared with the GM population (IMD 1: 41.5% vs 37.1%; p<0.0001). The clinic proportions were lower in comparison to the GM population decreasingly for those less deprived (IMD 5: 10.9% vs 12.0%, p<0.0001) (online supplemental appendix Figure 5).

For headache, a greater proportion of patients from the highest deprivation were seen in clinics relative to the GM population (IMD 1: 44.0% vs 37.2%, p<0.0001). While the least deprived were under-represented in clinic (9.4% vs 12.0%, p<0.0001) (online supplemental appendix Figure 6D). After adjusting for age, sex and LSOA, the occurrence of headache in clinic decreased with decreasing deprivation, relative to the most deprived group (IMD 1) (RRs (95% CI) IMD 2. 0.89 (0.81 to 0.97), p=0.01; IMD 3. 0.86 (0.78 to 0.94), p=0.0016; IMD 4. 0.79 (0.72 to 0.87), p<0.001; IMD 5. 0.74 (0.67 to 0.83), p<0.001) (figure 2D). When age-standardised, there was an increased likelihood of being seen in neurology GM clinic for females with headache and increasing deprivation (ASRRs (95% CI) IMD 1. 1.64 (1.61 to 1.68); IMD 2. 1.36 (1.32 to 1.40); IMD 3. 1.27 (1.22 to 1.33); IMD 4. 1.18 (1.13 to 1.23); IMD 5. 1.09 (1.03 to 1.14)) (online supplemental appendix Figure 7A).

For epilepsy, again, proportionally more people were seen in clinic from the most deprived areas compared with the GM population (IMD 1. 47.0% vs 37.2%, p<0.0001), while those in the least deprived areas were seen less (IMD 5. 8.2% vs 12.0%, p<0.0001) (online supplemental appendix Figure 6B). After adjusting for covariates, the occurrence of epilepsy in clinic also decreased with decreasing deprivation (RRs (95% CI) IMD 2. 0.84 (0.78 to 0.91), p<0.001; IMD 3. 0.72 (0.67 to 0.78), p<0.001; IMD 4. 0.63 (0.58 to 0.68), p<0.001; IMD 5. 0.57 (0.52 to 0.63), p<0.001) (figure 2B). When age standardised, epilepsy was seen more in clinic if male and from higher deprivation (ASRR (95% CI) IMD 1. 1.36 (1.32 to 1.39)) and females from higher deprivation (IMD 1. 1.16 (1.13 to 1.19)). There was a lower likelihood of being seen in clinic from less deprived areas (IMD 3–5) with epilepsy (online supplemental appendix Figure 7D).

For FND, proportionally more people were seen in neurology clinic from the most deprived areas compared with the GM population (IMD 1. 50.6% vs 37.2%, p<0.0001) and seen less, proportionally, from the least deprived areas (IMD 5. 6.6% vs 12.0%, p<0.0001) (online supplemental appendix Figure 6C). After adjusting for covariates, FND occurrences in clinic decreased with decreasing deprivation (RRs (95% CIs) IMD 2. 0.77 (0.71 to 0.84), p<0·001; IMD 3. 0.65 (0·59 to 0.71), p<0.001; IMD 4. 0.54 (0.49 to 0.60), p<0.001; IMD 5. 0.45 (0.41 to 0.50), p<0·001) (figure 2C). When age-standardised, there was a higher likelihood of being seen if female and from higher deprivation (ASRRs (95% CI) IMD 1. 1.78 (1.73 to 1.83); IMD 2. 1.28 (1.22 to 1.34); IMD 3. 1.15 (1.09 to 1.22); IMD 4. 0.93 (0·88 to 0·99); IMD 5. 0.72 (0.67 to 0.78)) (online supplemental appendix Figure 7B).

For demyelination, more patients were seen from the most deprived areas (IMD 1) overall, but they were under-represented relative to the GM population (33.7% vs 37.2%, p<0.0001). However, while the least deprived groups were seen less in clinic, they were over-represented relative to the GM population (12.9% vs 12.0%, p<0.0001) (online supplemental appendix Figure 6A). When adjusted for covariates, patients who were less deprived were more likely to be seen for demyelination (RRs (95% CI) IMD 2. 1.12 (1.01 to 1.24), p=0.03; IMD 3. 1.12 (1.00 to 1.24), p=0.04; IMD 4. 1.15 (1.04 to 1.28), p=0.01; IMD 5. 1.08 (0.96 to 1.21), p=0.20) (figure 2A). When age-standardised, demyelination had a higher likelihood of a neurology clinic appointment if female and decreasing deprivation (ASRRs (95% CI) IMD 1. 1.21 (1.15 to 1.28); IMD 2. 1.38 (1.29 to 1.47); IMD 3. 1.37 (1.27 to 1.48); IMD 4. 1.42 (1.32 to 1.52); IMD 5. 1.34 (1.23 to 1.45)) (online supplemental appendix Figure 7C).

For movement disorders, more patients were seen from the least deprived areas (IMD 5) relative to the GM population (13.6% vs 12.0%, p<0.0001) (online supplemental appendix Figure 6E). When adjusted for covariates, there was a lower likelihood of being seen if less deprived (RRs (95% CI) IMD 2. 0.90 (0.83 to 0.97), p=0.01; IMD 3. 0.83 (0.76 to 0.91), p<0.001; IMD 4. 0.80 (0.73 to 0.87), p<0.001; IMD 5. 0.83 (0.75 to 0.91), p<0.001) (figure 2E). When age-standardised, both sexes have a slightly higher likelihood of an appointment if from a more deprived area (ASRRs (95% CI) female 1.09 (1.05 to 1.13); male 1.21 (1.17 to 1.26)). But for less deprived areas, females are less likely to be seen (online supplemental appendix Figure 7E).

For neuromuscular disorders, more patients were seen from the least deprived areas (IMD 5) proportionally when compared with the GM population (13.2% vs 12.0%, p<0.0001) (online supplemental appendix Figure 6F). When adjusted for covariates, no significant differences were seen between IMD quintiles (RRs (95% CI) IMD 2. 0.92 (0.84 to 1.01), p=0.1; IMD 3. 0.92 (0.84 to 1.02), p=0.11; IMD 4. 0.91 (0.83 to 1.01), p=0.07; IMD 5. 0.91 (0.82 to 1.02), p=0.09) (figure 2F). When age-standardised and for both sexes, there is a slightly higher likelihood of being seen from the most deprived areas (ASRRs (95% CI): female 1.07 (1.03 to 1.07); male 1.14 (1.09 to 1.18)). For less deprived areas, females were less likely to be seen for neuromuscular disorders (online supplemental appendix Figure 7F).

## Discussion

Using an in-house preprocessing NLP pipeline, in conjunction with an open-source NLP tool, we have been able to successfully extract and categorise diagnostic data from GM neurology outpatient clinic letters and link this to sociodemographic data. To our knowledge, this is the largest UK outpatient neurology dataset analysed.

Our analysis shows that 43% of patients with neurological disorders are from the most deprived backgrounds in the GM region (online supplemental appendix Figure 5). The relationship between clinic attendance and deprivation was particularly pronounced for headache, epilepsy/seizures and FND (figure 2 and online supplemental appendix Figure 6). Similar results have been found with outpatient dissociative seizures in the UK,^18^ epilepsy in primary care in Wales and England^19 20^ and migraine in Scotland.^21^ It is unclear from our results whether the level of deprivation is a causative factor (social causation) of their neurological condition or whether there is a reduction in social status, for example, employment loss (social drift), consequently. Longitudinal data would be beneficial in this respect.

Higher numbers attending outpatient clinic from highest deprivation do not necessarily reflect better healthcare access. It may represent increased prevalence within that deprivation group, diagnostic and management difficulties in primary care due to poorer resources or overstretched services, and over-referring owing to complexity or multimorbidity. Delayed presentations can lead to increased severity requiring specialist care. Interestingly, for demyelination/inflammation, the opposite trend was seen with a higher likelihood of presenting to clinic from a less deprived background (figure 2). A systematic review of multiple sclerosis (MS) studies found that while some studies suggested a link between higher socioeconomic status and MS, overall, there were conflicting results, with no adjustments for possible cofactors, and so conclusions could not be drawn.^22^ MS disability has been associated with higher deprivation^23^ as well as reduced access to disease-modifying therapies.^24^ Lower representation for demyelinating conditions, such as MS, for the most deprived may be explained by earlier, more subjective symptoms such as numbness and tingling, being overlooked. The reduced impact of IMD on clinic attendance for movement disorders and neuromuscular disorders may be due to presence of objective findings, that is, tremor or weakness on examination, and clearer established clinical care pathways for these conditions.

Analysis of ethnicity was hampered by a lack of data recording for 16.5% of patients. While ethnicity coding is mandatory for many NHS datasets, patients can decline to answer leaving ‘Not Known’ or ‘Unknown’. There are also inconsistencies with different ethnicities recorded for the same patients in different datasets. While detailed ethnicities are recorded for data analysis, aggregation into five categories may lead to information loss for smaller, minority groups. Allowing for this, non-white ethnicities were under-represented in our patient cohort. Both Asian, black and mixed ethnicity patients are less likely to present to clinic for all diagnostic categories (online supplemental appendix Figure 3). For neuropsychological referrals in the GM region between 2014 and 2020, referrals were also proportionally higher for white patients versus non-white ethnicities.^25^ For patients from Asian, black and mixed ethnic backgrounds, barriers to referral and access to appropriate care may include under-referral linked to misattribution of symptoms, language and cultural barriers, socioeconomic factors and potential mistrust of healthcare services.

**Figure 3:**
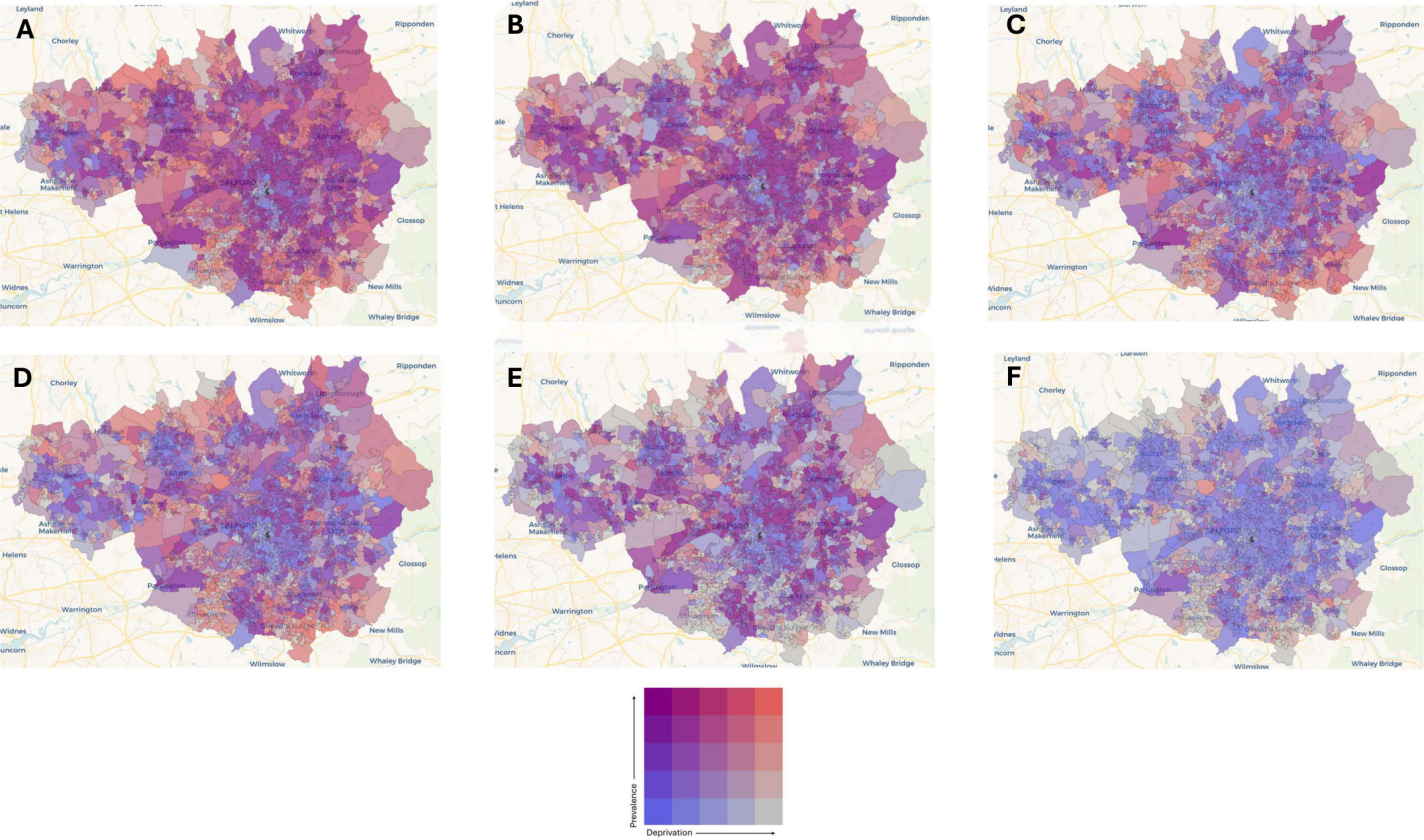
Bivariate choropleth maps for GM top six diagnostic categories looking at the relationship between IMD and prevalence rates for lower super output areas (1000–3000 population). (A) Headache. (B) Epilepsy. (C) Movement disorder. (D) Neuromuscular disorder. (E) FND. (F) Demyelination. Key: IMD (deprivation) is represented by a blue scale on the x axis and prevalence rates are represented by a red scale on the y axis. Areas with the highest prevalence for a neurological category, and highest deprivation are represented by the orange in the top right corner. FND, functional neurological disorder; GM, Greater Manchester; IMD, indices of multiple deprivation.

There are numerous advantages of obtaining large-scale outpatient diagnostic data using an automated pipeline with standardised coding. In clinic-based specialities, patient diagnostic data may not otherwise be captured via other medical reporting systems. Diagnoses are likely to be more refined, for example, ‘focal epilepsy with secondary generalisation’ rather than ‘epilepsy’. It also gives the opportunity to better understand the needs of the community by understanding the geographic burden. Using the data collected, interactions between variables of interest can be explored. Figure 3 demonstrates the relationship between IMD and neurological diagnostic category prevalence using bivariate maps. By identifying ‘hotspots’ where both outpatient prevalence and deprivation are high, resources could be allocated to high-need areas such as district hospital outreach, specialist nurses or community clinics.

The study has several limitations. A key limitation of the dataset is the extent of missing ethnicity data, which restricts the ability to accurately assess outcomes in minority ethnic populations. It is possible that missing data may be more frequent among individuals of non-white ethnicity. For the purposes of validation, we have only used the last neurology clinic letter and there is currently no temporal quality to the dataset. Additionally, only diagnostic data have been extracted thus far. Some diagnoses are not definitive (eg, possible Parkinson’s disease), which may require clinical judgement based on the context of the unstructured data contained within the letter. There is potential for misclassification bias where rule-based algorithms can misclassify when a diagnosis is not explicitly stated. Accuracy is better for frequent and well-defined diagnoses (eg, migraine, epilepsy) but poorer for heterogenous or rarer diagnoses. Finally, the data suffer from potential selection bias as those who have not been referred from the community or those who did not attend are currently unclear.

In the future, additional data of interest could be extracted from the body of letters, for example, treatments, diagnostics and comorbidities. Longitudinal trends and follow-ups can also be evaluated to determine potential disparities in care.

## Conclusions

Automated outpatient coding and demographic linkage in outpatient neurology can generate actionable data to identify unmet service needs and health disparities in access, supporting targeted deployment of resources and informed service planning.

## Supplementary material

10.1136/bmjno-2025-001532 online supplemental file 1

## Data Availability

No data are available.
