# Supplementary material for: Health inequalities in outpatient neurological conditions across a large UK urban population: a retrospective observational study using automated coding
Source: BMJ Neurol Open. 2026 May 20;8(1):e001532. doi: 10.1136/bmjno-2025-001532 (PMC13202089; doi:10.1136/bmjno-2025-001532)
Supplement: online supplemental file 1 [file bmjno-8-1-s001.pdf]

Appendix Figure 1. Cohort Flow Diagram. Greater Manchester neurology services refer to adults over 18 years old only.

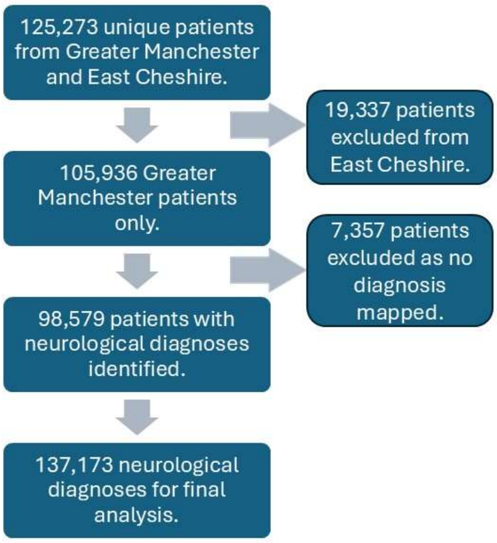

Appendix Figure 2. Histograms of age distributions for the top 6 diagnostic categories. Dashed red line represents the median age (only >18 years old seen in clinic). A. Demyelination/inflammation, B. Epilepsy/seizure, C. Functional/psychological disorder, D. Headache, E. Movement disorder, F. Neuromuscular Disorder.

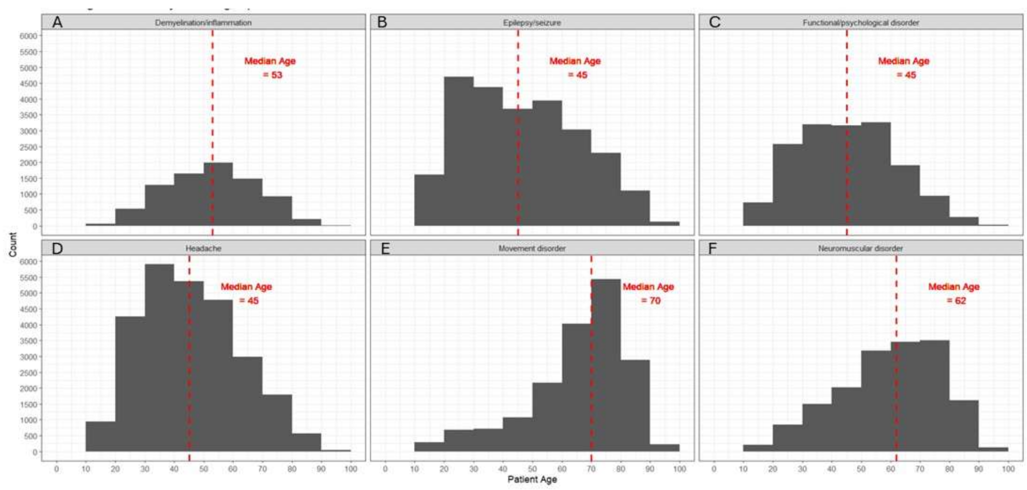

Appendix Figure 3. Greater Manchester (GM) outpatient neurology ethnicity clinic proportions compared to the GM population proportions (ONS census data 2021). Neurology outpatient clinic (blue dot) compared to the GM population (grey dot).

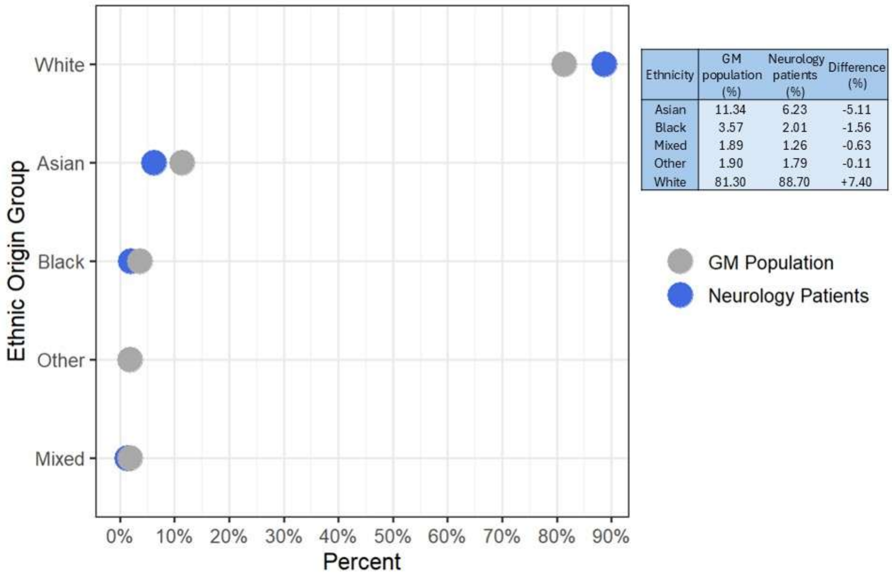

Ethnicity	GM population (%)	Neurology patients (%)	Difference (%)
Asian	11.34	6.23	-5.11
Black	3.57	2.01	-1.56
Mixed	1.89	1.26	-0.63
Other	1.90	1.79	-0.11
White	81.30	88.70	+7.40

Appendix Figure 4. Incidence Rate Ratios (IRR) of interaction between IMD and ethnicity.

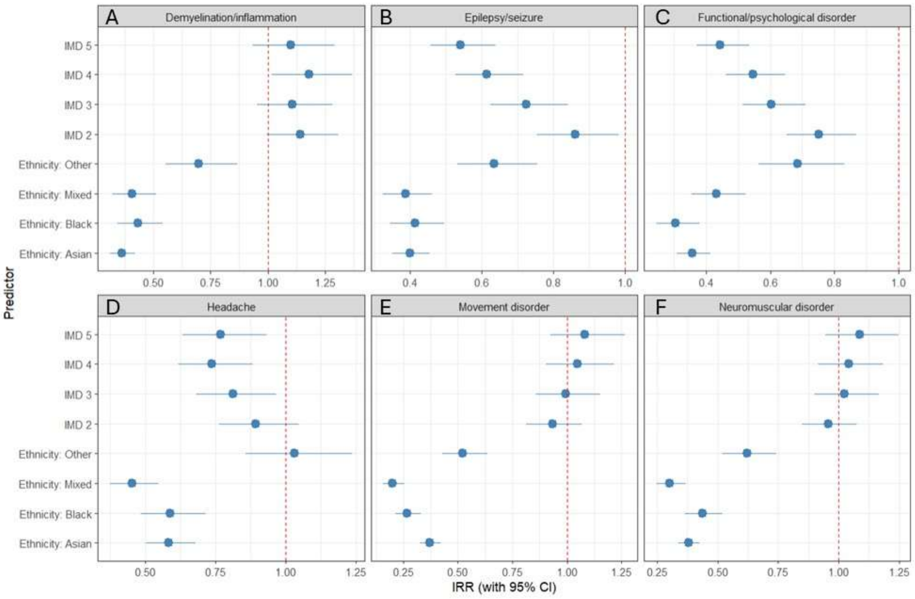

Appendix Figure 5. Greater Manchester (GM) neurology outpatient Index of Multiple Deprivation (IMD) quintiles versus the GM population. For patients seen in neurology clinic (blue dot) compared to the GM population (grey dot).

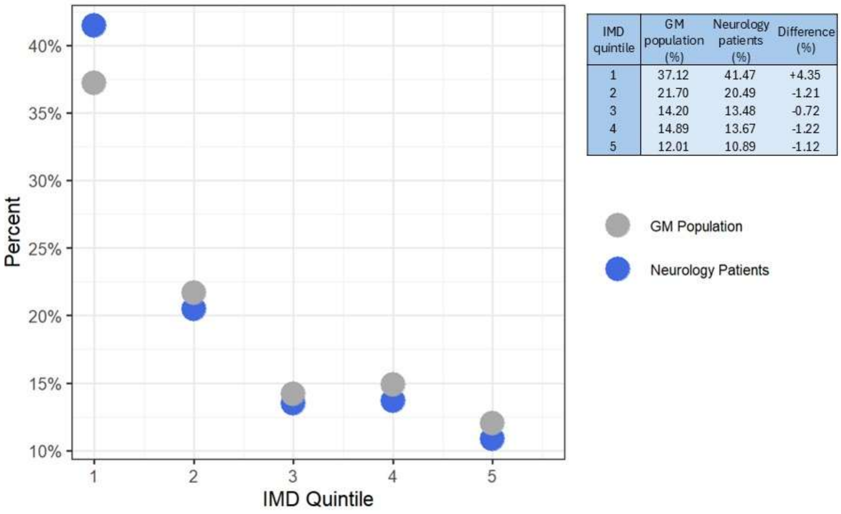

IMD quintile	GM population (%)	Neurology patients (%)	Difference (%)
1	37.12	41.47	+4.35
2	21.70	20.49	-1.21
3	14.20	13.48	-0.72
4	14.89	13.67	-1.22
5	12.01	10.89	-1.12

Appendix Figure 6. Index of multiple deprivation (IMD) quintiles for top six diagnostic categories: A. Demyelination/inflammation, B. Epilepsy/seizure, C. Functional/psychological disorder, D. Headache, E. Movement disorder, F. Neuromuscular disorder. IMD proportions are represented by a grey dot (Greater Manchester (GM) population), a blue dot (neurology clinic). For B, C, and D, there are a greater number of patients seen in IMD 1 (most deprived), compared to the GM population, but we see increasingly less for increasingly lower deprivation. For A, E, and F, relatively fewer are seen from more deprived areas (IMD 1) and higher numbers from the least deprived (IMD 5), compared to the population proportions.

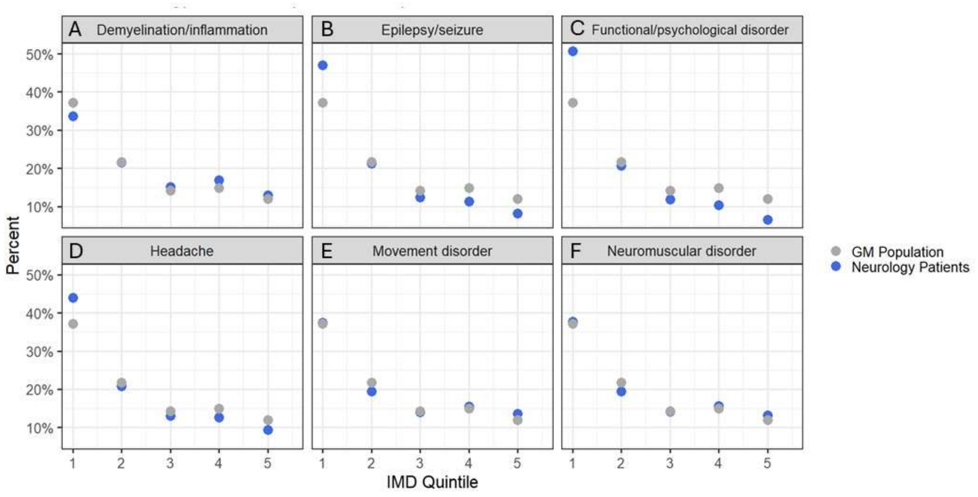

Appendix Figure 7. Age-standardised rate ratios (ASRR) for IMD and sex with 95% confidence intervals. For the dashed line, rate=1 (observed=expected). A: Headache, B: FND/ Psychological disorder, C: Demyelination/inflammation, D: Epilepsy/seizure, E: Movement disorder, F: Neuromuscular disorder.

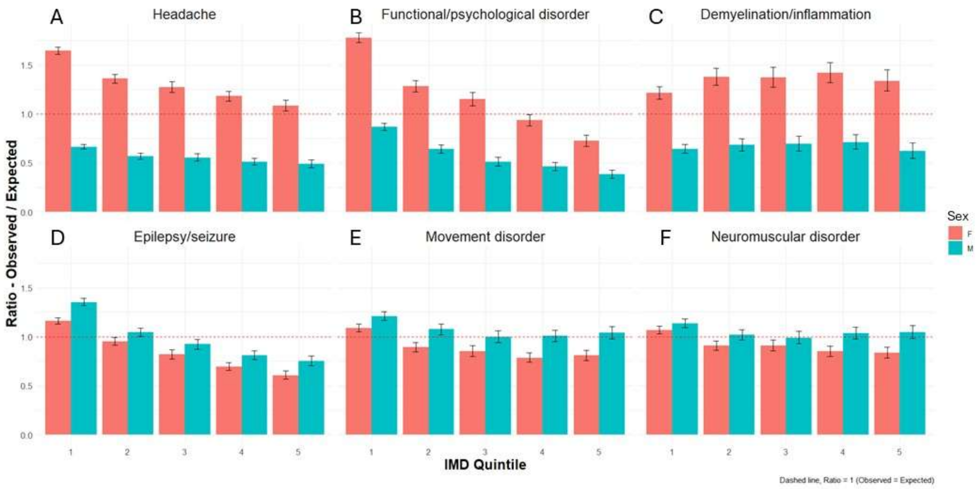

Appendix Table 1. Demographics of headline neurological disorders in outpatients in Greater Manchester. LQR (lower quartile range). UQR (upper quartile range). Ethnicity was categorised using standard NHS classifications: White, Mixed, Asian, Black, and Other ethnic group. The 'Other ethnic group' category included Arab and ethnicity who did not fall into the White, Black, Asian, Mixed ethnicity categories. Patients with missing data were coded as Unknown.

	**Headache**	**Epilepsy/ Seizure**	**Movement Disorder**	**Neuromuscular Disorder**	**Functional/ Psychological Disorder**	**Demyelination/ Inflammation**	**Spinal Degenerative Disease**	**Other**	**No diagnosis mapped**	**Suspected Neurological Disorder**	**Non-neurological**	**No definite neurological diagnosis**
**Age (years)**												
Median (LQR-UQR)	44 (33-57)	46 (31-61)	69 (58-77)	61 (48-73)	45 (33-57)	53 (41-64)	59 (48-71)	62 (45-74)	50 (35-64)	50 (36-64)	59 (46-71)	50 (38-63)
**Ethnicity (%(n))**												
White	68·3 (15,894)	75·0 (15,549)	78·1 (11,209)	79·4 (10,854)	75·1 (10,255)	84·7 (5,464)	79·0 (4,316)	76·9 (20,213)	70·6 (5,197)	71·0 (6,712)	72·0 (2,383)	67·1 (328)
Unknown	19·9 (4,634)	15·8 (3,263)	14·6 (2,092)	11·8 (1,612)	16·3 (2,221)	5·6 (363)	11·9 (650)	15·4 (4040)	19·0 (1,397)	20·2 (1,926)	18·4 (611)	19·6 (96)
Asian	6·7 (1,560)	5·1 (1,050)	4·6 (660)	4·9 (674)	4·4 (602)	4·9 (318)	5·5 (299)	4·3 (1,125)	5·1 (377)	5·1 (484)	5·7 (190)	6·3 (31)
Black	2·0 (462)	1·7 (351)	1·1 (154)	1·7 (235)	1·3 (178)	1·9 (120)	1·5 (84)	1·5 (395)	2·0 (148)	1·6 (153)	1·7 (55)	2·7 (13)
Mixed	1·3 (290)	1·2 (239)	0·6 (85)	0·9 (122)	1·4 (186)	1·3 (86)	0·9 (50)	0·8 (206)	1·2 (88)	1·0 (93)	0·9 (28)	1·8 (9)
Other Ethnic group	1·8 (423)	1·3 (272)	1·0 (147)	1·3 (182)	1·5 (206)	1·6 (103)	1·2 (63)	1·2 (301)	2·0 (150)	1·5 (146)	1·4 (46)	2·5 (12)
**Sex (%(n))**												
Female	71·5 (16,628)	47·9 (9,936)	48·5 (6,963)	49·2 (6,727)	68·1 (9,293)	67·5 (4,359)	55·6 (3,038)	48·8 (12,822)	54·1 (3,982)	61·4 (5,843)	54·0 (1,789)	59·5 (291)
Male	28·5 (6,635)	52·1 (10,788)	51·5 (7,384)	50·8 (6,952)	31·9 (4,355)	32·5 (2,095)	44·4 (2,424)	51·2 (13,458)	45·9 (3,375)	38·6 (3,671)	46·0 (1,524)	40·5 (198)
**Total**	23,263	20,724	14,347	13,679	13,648	6,454	5,462	26,280	7,357	9,514	3,313	489
